# Using Informatics and the Electronic Medical Record to Describe Antimicrobial Use in the Clinical Management of Diarrhea Cases at 12 Companion Animal Practices

**DOI:** 10.1371/journal.pone.0103190

**Published:** 2014-07-24

**Authors:** R. Michele Anholt, John Berezowski, Carl S. Ribble, Margaret L. Russell, Craig Stephen

**Affiliations:** 1 Faculty of Veterinary Medicine, University of Calgary, Calgary, Alberta, Canada; 2 Veterinary Public Health Institute, University of Bern, Bern, Switzerland; 3 Centre for Coastal Health, Nanaimo, British Columbia, Canada; 4 Community Health Sciences, University of Calgary, Calgary, Alberta, Canada; The University of Hong Kong, Hong Kong

## Abstract

Antimicrobial drugs may be used to treat diarrheal illness in companion animals. It is important to monitor antimicrobial use to better understand trends and patterns in antimicrobial resistance. There is no monitoring of antimicrobial use in companion animals in Canada. To explore how the use of electronic medical records could contribute to the ongoing, systematic collection of antimicrobial use data in companion animals, anonymized electronic medical records were extracted from 12 participating companion animal practices and warehoused at the University of Calgary. We used the pre-diagnostic, clinical features of diarrhea as the case definition in this study. Using text-mining technologies, cases of diarrhea were described by each of the following variables: diagnostic laboratory tests performed, the etiological diagnosis and antimicrobial therapies. The ability of the text miner to accurately describe the cases for each of the variables was evaluated. It could not reliably classify cases in terms of diagnostic tests or etiological diagnosis; a manual review of a random sample of 500 diarrhea cases determined that 88/500 (17.6%) of the target cases underwent diagnostic testing of which 36/88 (40.9%) had an etiological diagnosis. Text mining, compared to a human reviewer, could accurately identify cases that had been treated with antimicrobials with high sensitivity (92%, 95% confidence interval, 88.1%–95.4%) and specificity (85%, 95% confidence interval, 80.2%–89.1%). Overall, 7400/15,928 (46.5%) of pets presenting with diarrhea were treated with antimicrobials. Some temporal trends and patterns of the antimicrobial use are described. The results from this study suggest that informatics and the electronic medical records could be useful for monitoring trends in antimicrobial use.

## Introduction

Diarrhea is a common clinical presentation in companion animals [1]. The pathophysiology of diarrhea is complex, poorly understood and can involve a wide array of infectious and non-infectious etiologies [2], [3]. Clinical evaluation of ill animals directs the selection of diagnostic procedures such as parasite studies, microbiological examinations and/or toxin testing. Clinicians must weigh the cost of diagnostic procedures, the owner's willingness to pay for them and the time spent waiting for a result against the likelihood that the results of a diagnostic test will affect their therapeutic recommendations. This cost-benefit analysis often results in diarrhea in pets being managed by empirical therapy with antihelmintics and antimicrobials [4].

Infectious disease specialists advocate restricting antimicrobial use (AMU) to cases where there is evidence that AMU will result in improved clinical outcomes [3], [5], [6]. Warnings against indiscriminate AMU in animals are increasing because the consequences of AMU include antimicrobial resistance (AMR) with decreased efficacy of important antimicrobials against significant animal and human pathogens [7], [8]. In their closely shared environment, pets may be a source of antimicrobial resistant enteric bacteria or resistance genes for their owners [9]–[11].

Understanding the clinical management of common veterinary problems and patterns of AMU may provide the necessary exposure information to help interpret AMR trends, identify potential problem areas in prescribing practices and provide evidence-based practice guidelines for practitioners [12]–[16]. Collecting clinical management and AMU data at the veterinary patient level has not been legislated in Canada and remains a challenge in veterinary medicine in Canada [11], [17], [18].

The uptake of the electronic medical record (EMR) by companion animal practitioners provides an opportunity for accessing case management and AMU data. Informatics is “the application of information and computer science technology to public health practice, research and learning” [19]. Informatics has been applied elsewhere to text-based clinical records to describe disease-drug associations by physicians [20]. In this paper we used the EMR's from a participating practice network and explored text mining for accessing and analyzing the textual orders for diagnostic testing and AMU in the medical records.

The objectives of this study were to:

1. Apply and evaluate text-mining technology of EMR's to characterize the clinical management of diarrhea cases by companion animal veterinarians in a network of participating veterinary practices.
2. Describe the diagnostic management of diarrhea in companion animals and the proportion of cases for which there was documented evidence of an infectious process.
3. Describe the use of antimicrobials in the management of diarrhea cases.
4. Describe the temporal patterns of the use for each antimicrobial class used in the treatment of diarrhea cases for a 4 year period (January 1, 2007 to December 31, 2010).

## Materials and Methods

### Study area and data

The study area included 6 communities in the province of Alberta, Canada including: Calgary, Cochrane, Airdrie, Chestermere, Strathmore and Okotoks. A survey of all of the companion animal practices in the study area identified the practices that had completely computerized medical records and the same veterinary practice management software. Twelve of the 20 eligible practices agreed to participate in this project; a sample of convenience. A data sharing agreement was signed by each of the practice's managing partners and the author (Anholt). Approval from the University of Calgary Conjoint Faculties Research Ethics Board did not require permission from the pet owners.

A custom-built data extraction program was used to extract the anonymized electronic medical records (n = 428,783) from the veterinary practice management programs from January 1, 2007 to December 31, 2010. All records were stored in a secure data warehouse at the University of Calgary. The appointment schedule, medical notes (history, clinical exam, interpretations of diagnostic tests, assessment, differential diagnoses, and treatment) and prescription data for each case were combined into one free-text variable named ‘*Note*’, in the data file. Data was stored and managed using Microsoft Office Excel 2007 (Microsoft Corporation, Redmond, Washington) and Konstanz Information Miner 2.2.2 (Knime, http://www.knime.org). The features of the participating practices, data extraction and management of the warehoused data have been described elsewhere [21].

Linguistics-based text-mining software (QDAMiner3.1/WordStat6, Provalis Research, Montreal, QC), was used in this study. Text, in the form of individual words or phrases was organized into categorization dictionaries which were used to identify and retrieve cases. A categorization dictionary was applied to the ‘*Note*’ variable in the warehoused records to identify and retrieve records that met the case definition of any companion animal species (dog, cat, small mammal, bird, reptile) with clinical diarrhea or a description of feces consistent with diarrhea (n = 18,827 records). The case definition and the development, optimization and validation of the text miner to identify and retrieve records of diarrhea is further described in Anholt et al.[22].

Each of the 18,827 records represented a uniquely identified patient classified as having diarrhea, seen at a participating practice on a recorded date. After the initial visit, animals may have been hospitalized, returned for re-examination or there may have been a telephone consultation with the owners for the same complaint. To minimize repeated counts of the same case of diarrhea, all records of veterinary utilization (consultations, hospitalizations, laboratory results) for the same animal within 14 days of the initial visit were combined to represent one diarrhea case. There were 15,928 diarrhea cases in this study.

### Development of the categorization dictionary in the text miner

Text mining was used to identify and retrieve cases for which one or more of the following activities were recorded:

- diagnostic testing had been performed.
- an etiological diagnosis had been made.
- treatment with an antimicrobial had been initiated.

Case definitions were developed for diagnostic testing and etiological diagnoses to classify cases using the text miner and also by an external reviewer. For classification purposes a diagnostic test was a laboratory test that could either be performed in the practice by the animal health technologist or sent to an external veterinary laboratory. A case was classified as positive for diagnostic testing if any of the following diagnostic tests were recorded within the variable ‘Note’:

- Fecal flotations and fecal smears and using light microscopy that provided a morphological diagnosis of helminths, protozoa or bacteria.
- Enzyme-linked immunosorbent (ELISA) assays to identify canine parvovirus or *Giardia* spp. infections from fecal samples.
- Real time PCR tests were performed to screen fecal samples for canine distemper virus, canine coronavirus, canine parvovirus, *Clostridium perfringens* enterotoxin A, *Cryptosporidium* spp. *Giardia* spp., *Salmonella* spp., feline coronavirus, feline panleukopenia, *Toxoplasma gondii*, and *Tritrichomonas foetus*.
- Fecal bacteria culture was performed.

A case was classified as positive for etiologic diagnosis if a positive outcome for any of the diagnostic tests described above was recorded. The positive classification included imprecise morphological diagnoses of bacterial infections such as bacterial overgrowth and *Campylobacter*-type spp. as recorded by a veterinarian or technician.

Positive antimicrobial use cases were defined as those diarrhea cases that were administered, dispensed or prescribed antimicrobials for the management of the diarrhea signs.

To calculate the number of diarrhea cases required to assess the ability of the text miner to accurately classify the cases by each management activity (diagnostic testing, etiological diagnosis and antimicrobial treatment), the assumptions of the precision-based sample size calculation were: i) significance level, 0.05, ii) *a priori* estimate of the proportion, conservatively  = 0.5, iii) precision  = 0.1. The calculated number of cases positive for each activity required in the sample was 96. To reach the target of 96 positive cases in the sample required an estimate of the proportion of cases that would be positive for each activity. This was unknown and was expected to differ for each activity so a proportion of 0.20 was selected. The number of controls required was calculated using, N_controls_ = N_Cases_(1-Prev/Prev)  = 384 controls +96 cases  = 480 [23]. A sample of 500 records was randomly selected from the entire file of 15,928 diarrhea cases.

An experienced veterinarian clinician, blinded to the results of the text miner, reviewed all of the information contained in the extracted EMR's for the sample of 500 cases. The clinician reviewer classified each case as positive or negative for each of: i) laboratory diagnostics performed; ii) etiological diagnosis made; and iii) antimicrobial treatment. This served as the external standard.

We cross-tabulated the dichotomous results from the text miner and the external standard. The results for each case definition were summarized as the sensitivity and the specificity of the text miner's ability to correctly classify cases. The 95% confidence intervals for the sensitivity and specificity were also calculated (Exact method, Stata/IC 10.0, StataCorp, College Station, Tx). The cases that were improperly classified (false positives and false negatives) were reviewed to determine why they had been misclassified and if there were any opportunities to improve the text-mining classifier.

The sample of 500 diarrhea positive cases was categorized into three categories: i) no diagnostic testing performed, ii) diagnostic testing performed with a negative result or no result recorded; and iii) diagnostic testing performed with a positive diagnosis. Within each of the 3 categories the proportion of patients that were managed with antimicrobials was determined. Odds ratios (OR) and their 95% confidence intervals (CI) were used to quantify the difference between the odds of cases within each category receiving antimicrobials.

### Antimicrobial use trends

The text miner's categorization dictionary for antimicrobial use (described above) was then applied to all of the 15,928 diarrhea cases to classify cases that had been administered, dispensed or prescribed antimicrobials. Antimicrobial use was described by the class of antimicrobial used and by Health Canada's categorization of antimicrobial drugs based on importance to human medicine [24]. Co-occurrences of antimicrobial use were identified by the text miner and the antimicrobials used in combination were described.

We examined the temporal trends of the Category I (very high importance in human medicine) and Category II (high importance in human medicine) antimicrobials [24] for the 4 years of the study. For each month of the study, we determined the proportion of cases that had been treated with any antimicrobial and the proportions treated with each class of antimicrobial. The temporal trend for all antimicrobials combined and for each antimicrobial was examined by fitting a linear regression model to the data. The number of antimicrobial treated cases, normalized by the total number of diarrhea cases for each month, was the dependent variable and the month/year was the independent variable. If the antimicrobial use data fit the slope estimated by the linear regression (p<0.05), the proportions of cases treated with this antimicrobial were plotted as a function of time [25]. Further exploratory data analysis included data smoothing by: i) pooling the number of cases treated with each class of antimicrobial in each quarter of each year; and ii) plotting the results in scatterplots with quadratic overlays (Stata/IC 10.0).

## Results

### Text mining

Estimates of the text miner's ability to distinguish between cases that had diagnostic testing performed (sensitivity  = 70% and specificity  = 85.1%) and which had an etiological diagnosis made (sensitivity  = 72.4% and specificity  = 97.4), were relatively low. There were wide confidence intervals around sensitivity which indicated poor precision of the estimate (Table 1, Table 2). The primary reason the text miner performed poorly when classifying these cases was that the context was relevant to the classification of the case. For example, the word “parvo” was associated with a diagnosis, a differential diagnosis, a past diagnosis, a diagnostic test, a serological titer, a vaccine, and a recommendation or a warning to owners. Despite repeated efforts, it was not possible to improve the performance of the text miner to classify cases by the diagnostic test performed or their etiological diagnosis, so the text miner was not used for these purposes.

**Table 1:** From a random sample of 500 companion animal cases of diarrhea, the accuracy of the text miner for classifying the cases as positive or negative for ‘*had diagnostic testing*’ when compared to a manual review of the medical records serving as the external standard.

	External standard +	External standard -	Sum
**Text miner +**	63	61	124
**Text miner -**	27	349	376
**Sum**	90	410	500
	Sensitivity = 70.0% (95%CI, 59.4% - 79.2%)	Specificity = 85.1% (95%CI, 81.3% - 88.4%)	

**Table 2:** From a random sample of 500 companion animal cases of diarrhea, the accuracy of the text miner for classifying the cases as positive or negative for ‘*had an etiological diagnosis made*’ when compared to a manual review of the medical records serving as the external standard.

	External standard +	External standard -	Sum
**Text miner +**	21	17	38
**Text miner -**	8	454	462
**Sum**	29	466	500
	Sensitivity = 72.4% (95%CI, 52.8%–87.3%)	Specificity = 97.4% (95%CI, 95.5%–98.7%)	

In contrast, text mining classified cases that had been treated with an antimicrobial with high sensitivity (92.3%) and specificity (85%) when compared to a human reviewer (Table 3). The text miner misclassified cases if the name of the antimicrobial was not provided or improperly spelled, if the record contained information about past treatment or future considerations for treatment or if the pet was receiving antimicrobials but they were being used to treat a co-morbidity (not dispensed for diarrhea). Given the high sensitivity and specificity of the text miner for classifying cases with respect to antimicrobial use, it was used for the remainder of the analysis.

**Table 3:** From a random sample of 500 companion animal cases of diarrhea, the accuracy of the text miner for classifying the cases as positive or negative for ‘*had an antimicrobial administered, dispensed or prescribed*’ when compared to a manual review of the medical records serving as the external standard.

	External standard +	External standard -	Sum
**Text miner +**	215	40	255
**Text miner -**	18	227	245
**Sum**	233	267	500
	Sensitivity = 92.3% (95%CI, 88.1%–95.4%)	Specificity = 85.0% (95%CI, 80.2%–89.1%)	

### Diagnostic testing, diagnoses and antimicrobial use

**Figure 1:**
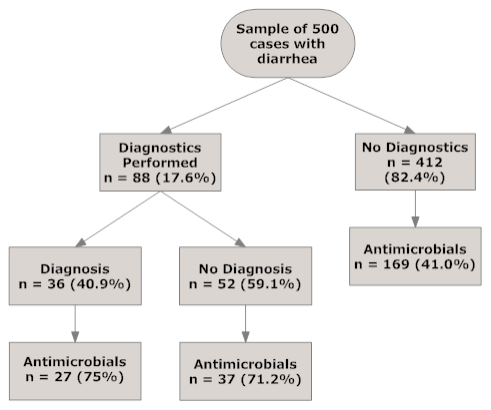
From a random sample of 500 companion animal cases with diarrhea, a flow diagram describing the proportion of cases that had laboratory diagnostics performed, had an etiological diagnosis made, and were administered, prescribed or dispensed antimicrobials.

As the text miner did not accurately classify cases that had laboratory testing performed or a diagnosis made, the results presented are from the manual review of the sample of 500 diarrhea positive cases only. The remaining diarrhea cases were not described by their diagnostic testing or etiological diagnosis. There were 88 cases (17.6%) in the sample of 500 diarrhea positive cases tested to identify an etiological diagnosis (Figure 1, Table 4). Fecal examinations (smears and/or floats) were performed in 56 of the 88 (63.6%) cases that underwent diagnostic testing; ELISA assays were run on 58 (65.9%) cases to identify canine parvovirus or *Giardia* spp.; multiple testing using a combination of fecal exams and ELISA tests was documented in 29 (33%) of those tested. Fecal cultures or PCR tests were each ordered in 1 (1.1%) and 3 (3.4%) of the cases respectively; all of which were negative. Thirty-six cases (40.9% of those tested, 7.2% of all cases) had a stated etiologic diagnosis in the EMR; all were prescribed an antihelmintic or antimicrobial medication. We inferred that given the management of cases with a positive result, that the veterinarians considered the findings to be relevant.

**Table 4:** Distribution of a sample of companion animal cases with diarrhea by the stated etiological diagnosis (n = 500).

Diagnosis	Number of cases (% of 500 cases)	% of diagnosed cases	Diagnostic test
All	36 (7.2)	-	
Helminths	1 (0.2)	2.7	Morphology
Coccidia	5 (1.0)	13.9	Morphology
Bacterial overgrowth	9 (1.8)	25	Morphology
*Campylobacter*-type	1 (0.2)	2.8	Morphology
Canine parvovirus	9 (1.8)	25.0	ELISA
*Giardia* spp.	11 (2.2)	30.6	Morphology or ELISA

Patients that had diagnostic procedures performed had more antimicrobials administered, dispensed or prescribed (72.7%) than patients that had no diagnostic testing performed (41%) (OR  = 3.8; 95% CI 2.2–6.7). There was little difference in the proportion of patients that were treated with antimicrobials and had a positive diagnostic test and those treated with antimicrobials and a negative diagnostic test (OR  = 1.2, 95% CI 0.4–3.6) (Figure 1). Two hundred and thirty-three of the 500 diarrhea cases (46.6%) received antimicrobials; none of the cases receiving antimicrobials were culture positive for bacteria (Figure 1, Table 4).

Text mining of the diarrhea cases (n = 15,928) identified 7400 (46.5%) cases that were administered, dispensed or prescribed antimicrobials. There were 8041 occurrences of AMU in the 7400 cases. The distribution of the antimicrobial classes used in the management of diarrhea positive cases is summarized in Table 5. Category 1 (very high importance to human health) antimicrobials were prescribed in most (87.1%) of the antimicrobial-treated diarrhea cases. Veterinarians prescribed more than one antimicrobial in 641 (8.7%) of all cases treated with an antimicrobial. Nitroimidazole plus a penicillin was the most frequent treatment combination (n = 346) followed by nitroimidazole together with first and second generation cephalosporins (n = 79), penicillins with fluorquinolones (n = 67), and nitroimidazoles in combination with fluorquinolones (n = 66).

**Table 5:** Distribution of antimicrobials used by the veterinary practices in the treatment of companion animal diarrhea cases (n = 15,928) in 2007, 2008, 2009 and 2010.

Health Canada Category [24]	Antibiotic class	Number of cases (% of 15,928 diarrhea cases)	% antimicrobial treated cases (n = 7400)
**Category 1 (Very High Importance)**	3^rd^/4^th^ Generation Cephalosporins	124 (0.8)	1.7
	Fluorquinolones	200 (1.3)	2.7
	Nitroimidazoles	5814 (36.5)	78.6
	Penicillin β – lactam inhibitors	310 (1.9)	4.2
	**Total for Category I**	**6448 (40.5)**	**87.1**
**Category II (High Importance)**	1^st^/2^nd^ Generation Cephalosporins	426 (2.7)	5.8
	Lincosamides	76 (0.5)	1.0
	Macrolides	124 (0.8)	1.7
	Penicillins	808 (5.1)	10.9
	Timethoprim-Sulpha	84 (0.5)	1.1
	**Total for Category II**	**1518 (9.5)**	**20.5**
**Category III (Medium Importance)**	Choramphenicol	5 (0.0)	0.1
	Sulphonamides	62 (0.4)	0.8
	Tetracycline	8 (0.1)	0.1
	**Total for Category III**	**75 (0.5)**	**1.0**

### Antimicrobial use temporal trends

**Figure 2:**
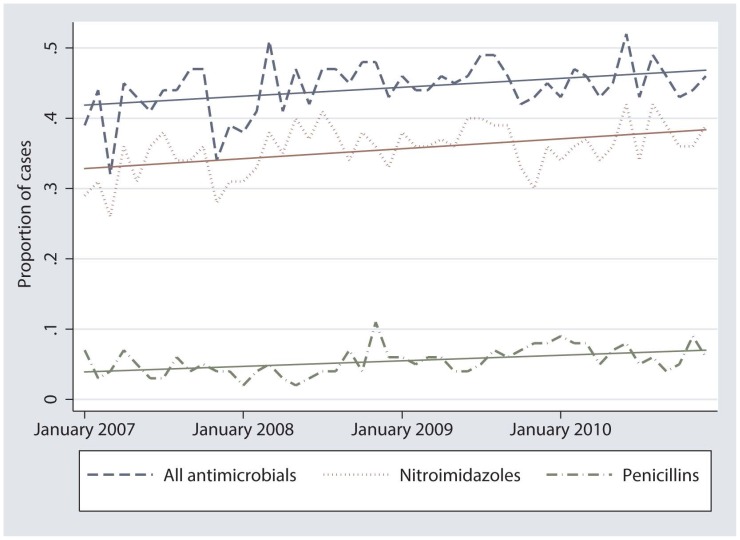
Changes in the proportion of companion animal diarrhea cases (n = 15,928) treated with any antimicrobial, nitroimidazole class and penicillin class from January 1, 2007 to December 31, 2010.

The linear regression analyses of ‘all antimicrobials’ (n = 7400), ‘nitroimidazole’ (n = 5814) and ‘penicillin’ (n = 808) were significant (p<0.05) and these variables were plotted against time (Figure 2). The graph and the slope coefficients (0.0002 to 0.0004) indicate a very small statistically significant, upward trend in the proportions of diarrhea cases treated with any antimicrobial and treated with nitroimidazoles and penicillins. The regression analyses of the remaining antimicrobials were not statistically significant.

**Figure 3:**
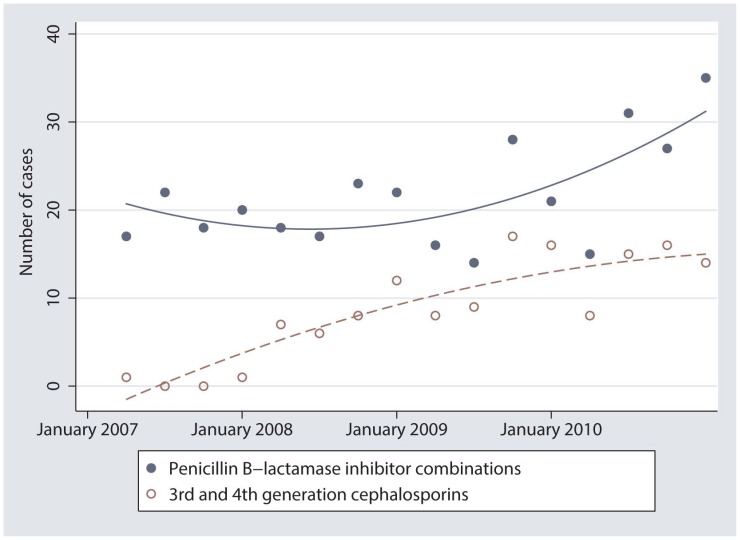
From 15,928 cases of companion animals with diarrhea, scattergrams of the counts of cases treated with B-lactam inhibitors and cephalosporins in each yearly quarter from January 1, 2007 to December 31, 2010.

Smoothed scatterplots of the quarterly counts of cases treated with 3^rd^/4^th^ generation cephalosporins and the penicillin β-lactamase inhibitor combinations showed patterns of antimicrobial use that were mirror images of each other (Figure 3). Scatterplots of the remaining antimicrobial class combinations did not show any recognizable patterns.

## Discussion

Results of the text mining methods used in this study varied depending on the variable of interest. Text mining results for AMU were relatively accurate because the documentation of antimicrobial treatments by veterinarians was usually explicit and unambiguous; the meaning of the words did not depend upon the context in which they were used. However, the language used to record diagnostic procedures and diagnoses was highly context specific and the linguistic-based text mining approach used in this study was unable to discriminate between the various meanings. It is possible that trained or rule-based text-mining software could more accurately distinguish these cases and is an area for future study [26], [27].

Most cases of acute (less than 14 days) diarrhea are mild and self-limiting and supportive treatment without a diagnosis is considered appropriate [2]. Therefore, it was not unexpected that less than 18% of the diarrhea cases in our study had diagnostic procedures performed. The recommended initial diagnostic approach to acute diarrhea is a fecal exam [28]. More than half of the diagnostic procedures in our study were fecal flotation and/or fecal smears. In animals with severe disease (febrile, dehydrated, hemorrhagic or persistent diarrhea) further efforts at establishing an etiological diagnosis are warranted [2], [28]. Animals in this study that were subjected to diagnostic laboratory testing were more likely to be given antimicrobials than those that were not tested regardless of the test results. This may indicate an assessment of more severe disease by the veterinarian although this judgment was not often explicitly stated in the medical record. Despite efforts to identify an etiological agent, a positive diagnosis was established in less than half of the cases undergoing diagnostic testing.

Giardiasis was the most frequent diagnosis in this study and antimicrobial treatment is usually recommended in *Giardia*-positive diarrheic animals [29]. However, *Giardia* spp. is commonly misdiagnosed in veterinary practice and most cases are self-limiting [30]. Antimicrobials are also recommended in the management of diarrhea in companion animals if there is a positive diagnosis of secondary bacterial overgrowth associated with inflammatory bowel disease or culture-confirmed primary bacterial infections of *Salmonella*, *Campylobacter*, *Clostridium* and enterotoxigenic *E. coli* [2], [4], [5], [29], if there is evidence of a breach in the mucosal integrity of the intestines (hemorrhagic diarrhea), or to manage the immunosuppressive effects of parvovirus [2], [4], [5], [28], [29]. Other authors argue that while antimicrobials are commonly used in cases with a confirmed culture or if there is evidence of hematochezia, there is little objective information as to whether they are needed in all cases [3], [5].

Our findings indicated that veterinarians commonly prescribed antimicrobials for diarrhea without any documentation that the animal's diarrhea had an infectious etiology. Empirical combinations of antimicrobial treatments was also common. Empirical antimicrobial use may lead to treatment failures and antimicrobial resistance [3], [4], [28], [29]. We found no post-prescription, pharmacoepidemiological studies evaluating empirical antimicrobial management of diarrhea in pets in the refereed literature.

Using the data extracted from medical records it was possible to detect changing trends in AMU. Despite increased AMR concerns [4], [6] there was evidence that nitroimidazole and penicillin use for the management of diarrhea in companion animals was increasing. Metronidazole (a drug of the Nitroimidazoles Class) was the most frequently prescribed antimicrobial and its use increased over the 4 years of the study. It is the drug of choice for anaerobic and microaerophilic bacteria (*Bacteroides* and *Clostridia*) and parasites (*Giardia* spp.) in animals [4]. In people it is important in the management of these pathogens and *Helicobacter pylori* [31], [32]. There are few therapeutic alternatives for these infections in people and so it is classified as a Category I antimicrobial [24]. Sensitivity testing for anaerobes is not routinely performed but treatment failures have been documented [32], [33] and the molecular basis for resistance has been established [31]. We found no papers documenting the transmission of metronidazole-resistant bacteria from pets to people.

The increase in the number of cases treated with 3^rd^ and 4^th^ generation cephalosporins in early 2008 coincided with the Canadian approval on May 30, 2007 and subsequent distribution of Convenia (Pfizer Animal Health, Kirkland, QC) later in 2007 [34]. Convenia is the trade name for cefovecin, a third generation cephalosporin. The increase in cefovecin use corresponded to a decrease in the use of penicillin β-lactamase inhibitor combinations. The indications for use are similar for the 2 classes of drugs so it is possible that one class was being used as an alternative to the other. Starting in the middle of 2009, the relationship appeared to be inverted and this trend continued until the end of 2010, the reason for which is unknown.

The results from this study suggest that informatics and EMR's could be useful for supporting evidence-based practice, and for monitoring trends in AMU and changes in veterinary prescription behavior following interventions to modify their use. Temporal trends and regional differences could prompt further investigations to explore why the observed trends were developing. Interventions such as confidential benchmarking by comparing AMU among veterinarians may serve to help veterinarians recognize problems and reduce AMU [35]. Analytical studies to see if there is an association between AMU in companion animals with diarrhea and the development of AMR in fecal microorganisms are indicated and informatics could provide the exposure data necessary to interpret AMR results.
